# Selenium: A Key Element in Inflammatory Bowel Disease

**DOI:** 10.3390/antiox14111299

**Published:** 2025-10-29

**Authors:** Francesca Gorini, Alessandro Tonacci

**Affiliations:** Institute of Clinical Physiology, National Research Council, 56124 Pisa, Italy; alessandro.tonacci@cnr.it

**Keywords:** selenium, selenoproteins, inflammatory bowel disease, Crohn’s disease, ulcerative colitis, oxidative stress, inflammation, immune system, gut microbiota, nanoparticles

## Abstract

Inflammatory bowel disease (IBD) is a multifactorial and complex condition of the gastrointestinal tract shaped by host genetics, immune dysregulation, gut microbiota and environmental determinants, with a steadily rising global prevalence. Although the etiology of IBD remains incompletely understood, chronic inflammation accompanied by oxidative stress, immune dysregulation, and gut dysbiosis is widely recognized as a hallmark of the condition. Given the frequent occurrence of undernutrition in IBD patients, the role of vitamins and micronutrients in modulating disease activity has been recently explored. Selenium (Se) is universally recognized as an essential trace element due to its diverse physiological functions, including potent antioxidant activity, anti-inflammatory effects, immunomodulatory properties, and the ability to influence gut microbial composition and diversity. This comprehensive review examines current evidence on the relationship between Se status and IBD, integrating epidemiological and experimental findings, elucidating the underlying biological mechanisms, and introducing Se nanoparticles, a viable therapeutic option using Se in IBD management.

## 1. Introduction

Inflammatory bowel disease (IBD), which primarily includes Crohn’s disease (CD) and ulcerative colitis (UC), is characterized by non-infectious chronic inflammatory conditions of the gastrointestinal tract possibly involving a complex interplay between genetics, environmental factors, gut microbiota alterations and dysregulation of the immune response [1,2,3]. In CD, skip lesions affecting any part of the gastrointestinal tract and clinical manifestations are variable and include diarrhea, abdominal pain, nausea, vomiting and weight loss [4]. Patients with UC that, unlike CD, is confined to the rectum and part of the colon or the entire colon, typically present with diarrhea, which can be accompanied by blood and other symptoms such as abdominal pain, urgency, tenesmus, and incontinence [5,6]. While CD is generally predominant in females, but with differences based on geography, males are more prone to developing UC than females, although the epidemiology of UC appears to vary with age [7]. Sex also influences other aspects of IBD, such as onset and clinical outcomes. Indeed, a recent meta-analysis revealed that males have a higher risk of mortality, and they are more frequently subjected to surgical interventions, whereas females report a worse quality of life, experiencing anxiety and depression [8]. Due to its relapsing-remitting course and potential associated complications, the treatment of IBD is lifelong and mainly relies on medications (amino salicylates, corticosteroids, immunomodulators, and biologics) for symptom control, although new therapeutic options (apheresis, cell and exosome therapies) have recently been discovered, aiming to ameliorate local mucosal inflammation and restore the normal mucosal structure [9,10]. Based on the more recent data, in 2019, the global age-standardized incidence rate of IBD was 4.97 per 100,000 person-years (with the highest value at 24.51 recorded in high-income North America) and the mortality rate was 0.54 per 100,000 person-years (in Eastern Europe at 0.64), corresponding to 405,000 new cases and 41,000 deaths, respectively [11]. Additionally, predictive models indicate that the age-standardized incidence rate is expected to remain relatively stable, while the age-standardized mortality rate is projected to decline over the next 30 years [10]. In Europe, approximately 0.4% of the population is affected by IBD, for a total of 2.5–3 million individuals [12]. This places a substantial burden on healthcare systems, comparable to that of major chronic diseases such as type 2 diabetes and cancer [11]. Approximately 50% of direct costs (estimated at 2.3–2.8 billion euros annually) are incurred during the first year following diagnosis due to hospital admissions and diagnostic, while the remaining costs are largely attributable to expenditures on biologic therapies [11]. Meanwhile, indirect costs, stemming from lost productivity due to absenteeism, presenteeism, delayed entry into the workforce, early retirement, premature death, and other intangible costs, also significantly contribute to the overall financial burden of IBD [13]. Considering the global increase in life expectancy and the resulting rising prevalence rates (nearly 1% in industrialized regions of Europe and North America), the costs of managing IBD will inevitably worsen in the future [13,14]. Indeed, the epidemiology of IBD appears to follow four epidemiological stages: the first, characterized by low incidence and prevalence; a subsequent phase of acceleration in incidence, but with low prevalence; stage 3 consisting of a stabilization or declining incidence with a concomitant increased prevalence; and the final stage, which occurs when prevalence reaches a plateau due to the higher mortality rate relative to incidence as IBD patients’ age [15].

Importantly, until the end of the 20th century IBD was regarded as a condition confined to early industrialized countries (Europe, North America, Oceania) [11,15]. However, over the past decade, emerging industrialized regions of Africa, Asia and Latin America, have witnessed a marked rise in IBD incidence, suggesting that, in addition to improved healthcare systems for disease surveillance and access to health professionals and medical technology, environmental determinants such as westernized diet and lifestyle, smoking, antibiotic use, and air pollution might substantially contribute to IBD development [11,15]. Although the etiology of IBD remains under investigation, current data suggest that a chronic uncontrolled immune response leads to inflammation of the intestinal mucosa [16,17]. This process is coupled with the generation of oxidative stress (a phenomenon resulting from an imbalance between production and elimination of reactive oxygen species—ROS in cells and tissues) that may involve the deeper layers of the intestinal barrier and even the systemic circulation [16,17]. Furthermore, IBD patients frequently experience micronutrient deficiencies, particularly during the active phase of the disease, due to accelerated intestinal transit, reduced appetite and intentional food avoidance driven by gastrointestinal symptoms and the desire to prevent relapses [18,19,20,21]. Selenium (Se) is an essential trace element to humans and recognized as one of the most powerful antioxidants [20]. Se has been shown to be vital for the functioning of the central nervous system and thyroid [21,22]. It has been associated with reduced risk of certain cancer types [23], prevention of cardiovascular disease, lower risk of all-cause and diabetes mellitus-specific mortality [24], and the preservation of male fertility [25]. Indeed, in the form of selenocysteine, Se is incorporated into selenoproteins that are directly involved in mediating the antioxidant response, mitigating inflammation, regulating the immune system and influencing the gut microflora, thus potentially playing a key role also in controlling intestinal inflammation [18,26,27]. While a few epidemiological studies have observed Se deficiency in patients with IBD, other authors could not detect significant differences in serum Se levels between affected subjects and healthy controls [28]. Furthermore, to date, only a very limited number of clinical trials have been published to investigate the consequences of Se deficiency, and these have been exclusively performed in patients with UC [29].

Therefore, in this comprehensive critical review we examined the epidemiological evidence dealing with the relationship between Se and IBD, analyzed the biological mechanisms potentially involved and discussed the future strategies as a promising avenue for prevention and therapeutic intervention in IBD management.

## 2. The Pathogenesis of Inflammatory Bowel Disease: An Overview

Although precise pathophysiology for IBD remains elusive, it has been widely acknowledged that its onset and progression involve a combination of host, genetic and microbial factors [30,31]. Notably, IBD reflects a breakdown in the delicate equilibrium between the host’s immune system and the gut environment, resulting in chronic inflammation of the intestinal mucosa [30,31].

The immune system plays a central role in this disruption. Intestinal immune cells, located in the intestinal epithelium alongside various specialized cell subtypes, are defective in CD [32]. Disruption of the intestinal epithelial barrier, which normally represents a selective and protective interface, leads to the condition known as “leaky gut”, characterized by increased permeability and exposure to luminal antigens [32,33]. Innate immune cells (dendritic cells - DCs, innate lymphoid cells, macrophages, natural killer (NK) cells, and neutrophils) become hyperactivated in IBD [34]. Macrophages, in particular, shift toward a pro-inflammatory M1 phenotype, releasing cytokines like interleukin (IL)-1β, IL-6, IL-12α, IL-23, and tumor necrosis factor alpha (TNF-α) [35]. Conversely, the anti-inflammatory M2 phenotype, which promotes tissue repair via IL-10, is diminished in IBD [35,36].

The adaptive immune system also plays a pivotal role. T helper (Th)1 cells, historically associated with CD, and Th2 cells, linked to UC, are both involved in disease progression [34,37,38]. More recently, Th17 cells have emerged as central players in IBD pathogenesis, with elevated levels found in both peripheral blood and intestinal mucosa of affected patients, resulting in exacerbation of inflammation and tissue damage [38,39]. Meanwhile, regulatory T cells (Tregs), which suppress Th17 responses and maintain immune tolerance, are reduced, leading to a decreased Treg/Th17 ratio [40]. In addition, patients with IBD display elevated systemic levels of immunoglobulin (Ig)G and IgA, reflecting substantial disturbances in humoral immunity [41].

Conventional immunosuppressive therapies (corticosteroids, immunomodulators, biologics), have demonstrated clinical efficacy but are associated with adverse events [42]. Emerging approaches such as mesenchymal stem cell therapy represent promising novel therapeutic avenues in modulating immune responses with fewer side effects [30,41,42,43].

In parallel, the gut microbiota undergoes significant alterations in IBD. Normally, the gut microbiota plays a crucial role in intestinal health by regulating the host’s innate and adaptive immune systems and energy metabolism, producing essential compounds such as vitamin K and short-chain fatty acids (SCFAs: acetate, butyrate, and propionate), and ultimately preserving the integrity of the intestinal mucosa from pathogenic attack [32,44,45]. In IBD, intestinal dysbiosis is marked by reduced alpha diversity (the metrics describing the species richness, evenness, or diversity within a sample [46]), and beta diversity (the measurement of similarity between two or more samples [46]), depletion of beneficial taxa (e.g., *Faecalibacterium prausnitzii*, *Roseburia* spp., butyrate-producing bacteria) [47,48,49,50,51,52,53,54], and enrichment of opportunistic pathogens like *Escherichia*/*Shigella* and *Ruminococcus gnavus* [52,54,55,56,57,58]. Gut dysbiosis worsens intestinal inflammation by activating innate and adaptive immune cells and impairing Treg function [30,59,60].

Metabolomic changes further exacerbate disease. Reduced SCFA levels impair inflammasome signaling and IL-18 production [45]. Altered bile acid profiles affect Treg differentiation via Farnesoid X Receptor (FXR) signaling, while FXR itself can modulate microbiota composition [61,62,63,64]. Tryptophan-derived metabolites such as indole-3-acetic acid and indole-3-aldehyde, which activate the aryl hydrocarbon receptor, are diminished in IBD, weakening anti-inflammatory pathways [65,66].

Therapeutic strategies targeting microbial balance such as probiotics, fecal microbiota transplantation, exclusive enteral nutrition, and nanodrugs like HABN, aim to promote epithelial repair and modulate microbiota composition [30,44].

Genetic susceptibility also plays a crucial role in IBD pathogenesis. Over 200 risk loci have been identified through genome-wide association studies, with stronger genetic contribution in CD than in UC [67,68,69]. Nucleotide-binding oligomerization domain-containing protein 2 (*NOD2*), the first susceptible gene identified in IBD, is involved in bacterial recognition and autophagy induction in DCs [68]. Notably, *NOD2* deficiency or mutations have been associated with dysbiosis, including an increased abundance of *Escherichia coli* and a reduction in *Faecalibacterium prausnitzii* [70,71]. Autophagy-Related 16-like 1 (*ATG16L1*) regulates autophagy in Paneth cells, and *ATG16L1T300A* variant alters microbial composition [72,73,74]. Caspase Recruitment Domain 9 (*CARD9*) gene, expressed in DCs, neutrophils and macrophages, influences immune responses and microbiota balance [72,75]. Common *CARD9* variants are associated with a pro-inflammatory phenotype enhancing immune response against pathogens, whereas rare variants exert a protective effect by dampening the immune response [30,75]. Furthermore, *CARD9*-deficiency disrupts tryptophan metabolism and delays colitis recovery [30,75,76]. Tumor Necrosis Factor Superfamily Member 15 (*TNFSF15*) encodes TNF-like cytokine 1A (*TL1A*), which promotes cytokine secretion and bacterial clearance [77,78]. *TNFSF15* polymorphisms have been significantly associated with IBD, and this relationship is influenced by ethnicity [72,79,80,81,82,83] (Table 1).

Monogenic IBD, typically presenting early or very early onset (before 5 years or even before 2 years, respectively), results from rare mutations affecting immune regulation, microbial clearance, or epithelial integrity [30,84,85]. Over 80 genes with Mendelian inheritance patterns have been implicated, including *IL-10*, *IL10RA*, *IL10RB*, *FOXP3*, *XIAP*, *CYBA*, *CYBB*, *TTRC7A* [30,85,86]. Gene therapy via hematopoietic stem cell transplantation targeted treatment for select cases of monogenic IBD, particularly when gene expression is restricted to bone marrow-derived cells [87].

Epigenetics, the set of heritable processes that influence phenotype without altering underlying DNA sequence, add another layer of complexity, accounting for a portion of the disease’s heritability [84,88]. In IBD, hypermethylation of genes like *IFN-γ* and *PAR2* correlates with disease severity, while *RUNX3* contributes to immune activation by enhancing Th1 and Th2 responses [89,90]. Additionally, histone-modifying enzymes such as histone deacetylase and protein arginine methyltransferase 2 are frequently overexpressed, promoting pro-inflammatory gene expression and suppressing regulatory pathways [91,92].

Together, these insights underscore the dynamic nature of IBD pathogenesis, where genetic, microbial, and immunological factors converge. They also highlight emerging therapeutic strategies aimed at restoring immune balance and microbial harmony, paving the way for more personalized and effective disease management.

## 3. Essential Elements and Inflammatory Bowel Disease

Among the various environmental determinants, diet is considered a major contributor to the etiopathogenesis of IBD, playing a central role in modulating intestinal inflammation, through its influence on microbiota balance, epigenetic regulation, and immune response [1,93]. While obese individuals are at higher risk of developing IBD (representing around 20% of patients [94]), malnutrition in IBD has been linked to worse prognosis and quality of life as well as increased hospitalization and mortality rates [95]. However, the role of diet in disease progression remains largely underestimated and no official nutritional guidelines currently exist for managing active IBD, as no specific dietary pattern has consistently been shown to reduce flare rates in adult patient [95,96,97]. Nonetheless, patients may be advised to follow a Mediterranean-style diet, characterized by a high intake of fresh fruits and vegetables and a low consumption of red and processed meats [97]. Certain dietary components, such as fiber from fruits and vegetables, have demonstrated beneficial effects in preserving the proper functioning of the intestinal barrier [94,98]. In recent years, growing interest has been directed toward micronutrients like vitamins A, C, D, and E, and trace elements such as zinc (Zn), copper (Cu), iron (Fe), and Se due to their anti-inflammatory and immunomodulatory properties, potentially resulting in beneficial effects in IBD [1,99].

Zn is the second most widely distributed trace element in the human body after Fe [100] and in its divalent ionic form, plays a pivotal role in numerous physiological processes, including the catalytic activity of over 300 enzymes, the structural stabilization of proteins and enzymes such as the zinc finger proteins, and the regulation of signaling pathways involved in diverse biological functions [100,101,102]. Zn also modulates several aspects of the immune system, influencing the development and function of innate immune cells, including neutrophils and NK cells, as well as the activation and proliferation of T lymphocytes [103,104]. Zn absorption, which primarily occurs in the small intestine, is influenced by multiple dietary factors: Zn intake itself (with maximal absorption efficiency observed under low-Zn conditions [105]), dietary protein (which enhances Zn absorption [106]), phytate (a phosphorus storage compound in plants and the most potent inhibitor of Zn absorption [107]), and Fe, which reduces Zn uptake when consumed at high doses [108]. Recent estimates suggest that approximately 17% of the global population is at risk of inadequate Zn intake, with prevalence reaching up to 25% in certain regions of Africa [109]. Inadequate level of Zn may lead to immune impairment, with reduced number of NK cells and mature T lymphocytes, inflammation, with increased secretion of the pro-inflammatory cytokine IL-6, and deterioration of the diversity of intestinal microflora [100]. Zn deficiency is particularly common in patients with CD and UC due to chronic diarrhea and intestinal inflammation [104,110,111]. Zn deficiency has been associated with an increased risk of adverse outcomes including hospitalizations, surgeries, and disease-related morbidity in patients with IBD [112]. Conversely, Zn normalization in these patients improves disease activity [112].

Cu, existing in two oxidation states (Cu^+^ and Cu^2+^), serves as an essential catalytic cofactor in a broad range of biological processes, including mitochondrial respiration, antioxidant defense, and the synthesis of methionine and melanin [113,114]. Cu homeostasis is tightly controlled at the cellular level by a complex network of Cu-dependent proteins, including cuproenzymes, Cu chaperones, and membrane transporters, thereby maintaining systemic concentrations within a narrow range [113]. Excess Cu levels are responsible for Wilson disease, Alzheimer’s disease and Huntington’s disease, as well as various types of cancers [115]. On the other hand, in addition to causing anemia, peripheral and optic neuropathy, and myelopathy, Cu deficiency impairs innate and adaptive immune responses [116]. Similarly to Zn, Cu deficiency is frequently observed in IBD but, in contrast to Zn, Cu-insufficient patients tend to exhibit lower levels of C-reactive protein (CRP) and fecal calprotectin (FCP), as Cu concentration typically rises during the acute-phase response [117,118]. This suggests that systemic inflammation influences Cu and Zn homeostasis in opposite directions, thereby contributing to an elevated Cu/Zn ratio [117].

Fe is a major micronutrient in the human diet and an essential trace element involved in key physiological processes, including the synthesis of hemoglobin and myoglobin, the production of cytochrome enzymes and DNA, and mitochondrial function [119]. Once ferric iron (Fe^3+^) is reduced to its ferrous form (Fe^2+^) by the acidic environment of the stomach, it is primarily absorbed in the duodenum and upper jejunum and then stored in the liver, kidneys, blood serum, spleen, and bone marrow [104]. Fe homeostasis is primarily regulated at the level of intestinal absorption by hepcidin, a peptide hormone synthesized in the liver [119,120]. While Fe excess promotes the generation of ROS, leading to tissue and organ damage [121], Fe deficiency may result in microcytic anemia, which is one of the most common extraintestinal manifestations of IBD [104,122]. In patients with IBD, the primary cause of anemia is Fe deficiency, which may arise from several factors, including chronic blood loss and/or reduced Fe intake [123]. Notably, treatment with Fe supplementation in patients with IBD and iron deficiency anemia has been associated with reduced disease progression and IBD-related hospitalizations in more than 10% of cases [123,124].

Se, in particular, an essential element with diverse physiological functions, has been proven to support intestinal health and alleviate the severity of intestinal disorders, including IBD [125].

In the following sections, we will explore the key characteristics of Se and its biologically active forms, selenoproteins, in terms of their dietary sources, bioavailability, absorption and metabolism. We will examine their biological effects, the evidence currently available on their association with IBD and the potential applications of Se-based interventions in disease management.

## 4. The Role of Selenium in Inflammatory Bowel Disease

### 4.1. Selenium: An Overview

In nature, Se primarily exists in three forms—elemental, inorganic, and organic—of which elemental and inorganic Se (i.e., selenide, selenite, selenate) are characterized by low availability [126]. In contrast, organic Se compounds, in the form of selenoamino acids, such as selenocysteine (SeCys) and selenomethionine (SeMet), have greater stability and bioavailability [126,127]. In humans, Se intake is primarily derived from dietary sources, with negligible contribution from water and air [127]. The Se content of foods is strictly dependent on geographical factors, given the large variability in soil Se concentration across countries and even within regions of the same country (as occurs in China), with Se concentrations ranging from 0.1 to 100 mg/kg [104,126,128]. It is estimated that over 40 countries encompass areas with low Se concentrations, and between 0.5 and 1 billion people worldwide are affected from Se deficiency [129,130]. Therefore, although Se in plant-based foods (e.g., Brazil nuts, cereals) tends to be more bioavailable, animal-based foods, such as meat, milk, dairy products and eggs, represent a superior source when animals are raised on Se-supplemented feed or grazed on Se-fertilized pastures [127,131,132]. Similarly, fish and shellfish have a high Se content thanks to Se-rich marine waters [126]. In animal tissues, SeCys is the predominant chemical form, while SeMet is found in both plant and animal-derived foods [132]. Selenite, a form of inorganic Se, is rarely present in natural foods and is primarily introduced through supplementation [133].

The total Se content in the human body ranges from 3 to 20 gr depending on geographical area and dietary habits, with the liver and skeletal muscle serving as the major storage organs [127,134]. Once absorbed in the duodenum, in the enterocytes Se is metabolized into hydrogen selenide (H_2_S) via the trans-selenation pathway through cystathionine γ-lyase or via reduction by thioredoxin (TRX) reductases and glutathione system, depending on its dietary chemical form [126,135,136]. In the liver, H_2_S is converted to selenophosphate that is then incorporated into selenoproteins in the form of SeCys [132]. To date, 26 selenoproteins—characterized by the presence of SeCys residues in their active sites—have been identified in humans, mediating physiological functions and biological effects of Se [125,126]. Most selenoproteins function as antioxidants and participate in redox regulation, others are implicated in thyroid hormone metabolism, Se transport and storage, calcium metabolism, glucose and lipid metabolism, selenophosphate synthesis, myogenesis, protein folding and AMPylation, cell proliferation and apoptosis [127,135]. Selenoprotein P (SEPP1), which accounts for more than 50% of circulating Se, transports Se to other organs and tissues for incorporation into proteins [128,132]. In case of excess Se intake, the element is either stored in liver reserves as glutathione peroxidase 1 (GPX1) or converted into selenosugars or selenium ions for excretion [126]. Se is mainly excreted via urine, while unabsorbed dietary Se, once incorporated into bile, is eliminated through feces [128].

In assessing Se status, Se concentration in blood (whole blood, plasma or serum) is considered the most reliable biomarker at both individual and population levels, as these matrices reflect short-to-medium term exposure [137]. Similarly, urine also serves as a useful biological matrix for evaluating recent Se intake and can be effectively employed in population-level surveillance of Se status, appropriately adjusting for creatinine, osmolality, and specific gravity [137,138]. Toenails, non-invasive matrices widely used in epidemiological studies thanks to the easy collection, slow rate of growth and refractoriness to external contamination, provide retrospective insights into Se status over a period of approximately 3 to 12 months prior to sampling [139]. In particular, Se content in toenails is positively correlated with SEPP1 and organic forms of Se, while showing an inverse relationship with inorganic Se forms such as selenite and selenate [140].

The daily dietary Se intake recommendations are not standardized at the global level [126]. In 1996, the World Health Organization established a maximal daily safe dietary intake of 400 µg, recommending a dietary intake of 40 µg/day in adult men, sufficient to achieve full plasma expression of GPX3 (corresponding to Se plasma/serum concentration of 90 μg/L [141]), and assuming a variability of dietary Se intake of around 16% [142]. Although GPX3 activity serves as a useful biomarker of Se status, it reaches a steady state at Se levels insufficient to saturate SEPP1, which is considered a more reliable indicator in reflecting saturation of the functional Se body pool [143]. In 2014, the European Food Safety Authority (EFSA) set an adequate intake of Se at 70 μg/day for adults, corresponding to plasma Se concentrations between 110 and 125 g/L, levels associated with maximal SEPP1 expression [143,144]. EFSA recently established a tolerable upper intake level for Se of 255 μg/day for both adult men and women, including pregnant and breastfeeding women [145]. Based on current dietary patterns, it is unlikely that European adults would exceed this intake unless consuming Se supplements or large amounts of Brazil nuts [145]. Severe Se deficiency, characterized by reduced systemic selenoprotein expression, has been linked to oxidative stress and an increased risk of conditions such as Keshan disease, Kaschin–Beck disease, and White Muscle Disease [126]. Conversely, chronic Se overexposure may lead to toxicity, known as selenosis, with clinical manifestations including anemia, fatigue, growth retardation, hair loss, and liver cirrhosis [145,146]. A cross-sectional study performed on subjects living in a seleniferous area in India, reported nail and hair alterations at a median serum concentration of 250 μg/L [147]. Furthermore, findings from a randomized, placebo-controlled trial indicate that an average Se intake of 330 μg/day over a median duration of 5.5 years (comprising approximately 130 μg/day from the background diet and 200 μg/day from L-selenomethionine supplementation) was associated with an increased risk of developing alopecia and dermatitis [148]. Notably, recent studies also suggest that excessive Se levels are positively associated with type 2 diabetes, hypertension, cardiovascular disease, cancer, and neurodegenerative diseases such as amyotrophic lateral sclerosis, Alzheimer’s and Parkinson’s disease [99,149].

**Figure 1:**
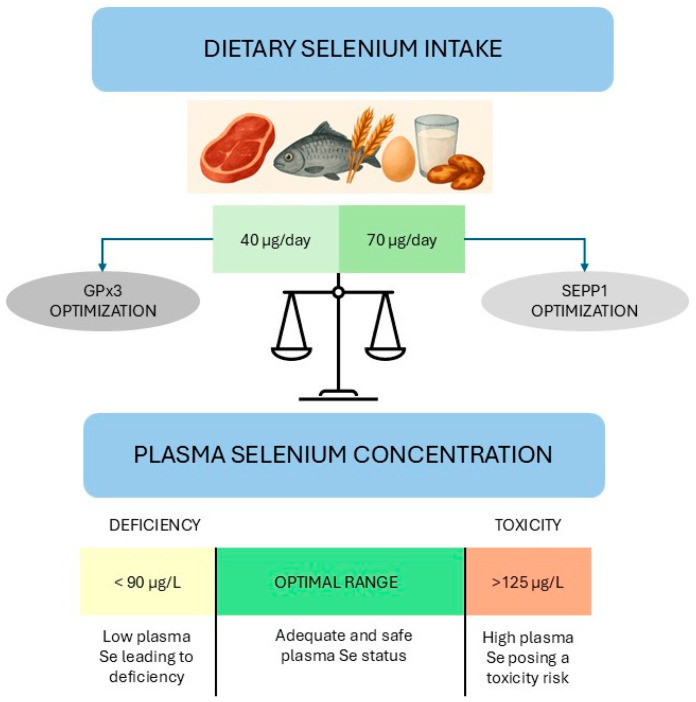
Interconnections between dietary selenium intake, selenium biomarkers, plasma selenium levels. Abbreviations: GPX3: glutathione peroxidase 3; Se: selenium; SEPP1: selenoprotein P. Image partly generated using the Microsoft 365 Copilot AI.

Collectively, these findings underscore the existence of a narrow range for plasma Se concentration underlying adequate and safe Se status [26] (Figure 1). Nonetheless, the identification and validation of reliable biomarkers of exposure and effect would enable more refined hazard characterization [145]. Moreover, current data on the consumption of Se-fortified foods and Se supplements remain limited; if systematically collected alongside comprehensive food composition data within a robust database, such information could serve as a valuable resource for monitoring Se intake at the population level and improving intake estimates [145].

### 4.2. Selenium and the Inflammatory Bowel Disease: Epidemiological Evidence

Nutritional status of IBD patients is commonly imbalanced, with malnutrition frequently arising from reduced food consumption due to gastrointestinal symptoms (abdominal pain, diarrhea, nausea, vomiting), prolonged restrictive diets, malabsorption, and adverse effects of pharmacological treatment [150,151,152]. Approximately 30–75% of patients with CD and 18–62% of patients with UC experience nutritional inadequacy [151]. Undernutrition is often present at the time of diagnosis, throughout the disease course and even during the periods of remission [150]. Consistently, micronutrient and vitamin deficiencies are prevalent in patients with IBD, with degree of deficiency depending on disease severity, dietary intake, supplementation, and medication use [151]. Among micronutrients, Se deficiency is a common finding in IBD patients, although the exact prevalence of Se depletion in IBD remains undefined [151]. A limited number of studies have explored the relationship between Se status and IBD, and with somewhat conflicting findings due to heterogeneity across studies [28] (Table 2). A Korean study conducted on 83 subjects diagnosed for IBD between 2013 and 2015, and age- and sex-matched controls, showed that 30.9% of patients had serum Se levels below the reference threshold of 95 µg/L [153]. Furthermore, female sex and low albumin levels emerged as independent risk factor for Se deficiency in multivariate logistic regression analysis [153]. A retrospective study involving 135 Chinese consecutive patients (94 men and 41 women) hospitalized for CD between May and December 2020, revealed a significant correlation between serum Se concentration and multiple inflammatory parameters, including CRP, the Crohn’s disease activity index (CDAI,) erythrocyte sedimentation rate, Harvey-Bradshaw index (HBI), platelet count, simple endoscopic score for Crohn’s disease (SES-CD), the latter being a reliable biomarker of CD severity [154]. The strongest correlation was observed with endoscopic evaluation [154]. However, these associations lost statistical significance in multivariate analysis, whereas the nutritional indicators albumin and folic acid independently maintained strong positive correlations with Se status, suggesting that low levels of these markers may serve as predictors of Se deficiency in CD patients [154]. Additionally, serum Se levels were significantly lower in patients with severe disease compared to those with milder forms, further supporting a link between Se status and CD activity [154]. A systematic review including six studies evaluating Se insufficiency in CD, reported variable findings: low blood Se levels in 5% (60 µg/L), 50% (72 µg/L) and 62% (47 µg/L) of patients in clinical remission across three studies; lower blood Se concentration in 13% of patients compared to healthy controls in one study; no significant associations between Se levels and disease activity in two studies [155]. A broader systematic review and meta-analysis encompassing 20 studies published between January 1980 and December 2020 for a total of 1792 patients with CD or UC and 1648 controls, demonstrated that IBD is significantly associated with reduced blood Se levels [28]. Sensitivity analyses accounting for variables such as detection method, geographical region, biological matrix, study design, participant age, and conducted separately for CD and UC, confirmed the overall findings results, with more consistent results observed in the CD subgroup [28]. Chalcarz et al. [156] reported significantly lower Se levels in Polish patients with IBD (CD: *n* = 54, 64.79 µg/L ± 12.15 µg/L; UC: *n* = 46, 68.61 µg/L ± 11.43 µg/L) compared to controls (*n* = 100, 90.52 µg/L ± 12.00 µg/L). Se concentration significantly declined with increasing disease severity in both IBD groups, although with different patterns: in CD, the correlation was statistically significant regardless of the criteria adopted for disease severity (CDAI, HBI, SES-CD), whereas in UC the association was significant only when assessed using the Clinical Activity Index scale [156]. In UC patients, Se deficiency was also independently associated with elevated leukocytes and erythrocyte counts and reduced bilirubin levels in the multivariate stepwise linear regression analysis, supporting the potential utility of Se as a predictive biomarker of disease activity [156]. Brownson and co-authors evaluated circulating levels of seventeen micronutrients in 216 English patients with IBD (127 with CD, 89 with UC, median age = 43 years) undergoing biologic therapy [19]. Although Se deficiency was observed in only 6.5% of subjects, plasma Se levels showed significant inverse correlation with CRP and FCP, and a positive association with serum albumin [19]. Over a 12-year follow-up period, Se deficiency was linked to increased risk of treatment with corticosteroids, clinical flare of disease and surgical intervention [19]. However, these associations were not significant in a small subset of patients with normal levels of disease biomarkers (i.e., CRP, FCP and albumin) [19]. Among UC patients clinically stable in the 3 months preceding the micronutrient assessment, low Se levels remained significantly associated with steroid requirement over the subsequent 12 months, even after adjusting for confounding factors (body mass index—BMI, CRP, FCP, albumin, hemoglobin, drug levels), whereas the association with clinical exacerbation was lost, indicating however that this effect is primarily driven by systemic inflammation [19]. A Chinese case–control study enrolling 80 CD patients and 45 healthy volunteers (aged 14–78 years) between July 2022 and November 2022 confirmed reduced serum Se concentration in CD patients compared to controls [157]. Consistent with findings by [154], Se levels were positively correlated with nutritional biomarkers BMI and albumin, and negatively associated with CDAI and CRP, reinforcing the link between Se deficiency and CD severity [157]. Importantly, the study also Se-related alterations in gut microbiota diversity and composition [157]. All α-diversity indices were significantly lower in fecal samples from Se-deficient patients than in those with normal levels, while beta diversity revealed substantial differences between the two groups [157]. At the phylum level, Se deficiency was associated with decreased abundance of Firmicutes, Bacteroidetes, Verrucomicrobia and increased levels of Actinobacteria and Proteobacteria [157]. Genus-level analysis showed reduced richness of *Bacteroides*, butyrate-producing *Lachnospieraceae*, *Faecalibacterium* and *Phascolarctobacterium* and higher richness of *Escherichia-Shigella*, *Fusobacterium* and *Morganella* in the Se-deficient group, suggesting an interaction between Se status and gut microbiol ecology [157].

Taken together, current epidemiological evidence supports the observation that IBD patients, particularly those with CD, exhibit significantly lower blood Se levels compared to healthy individuals. While Se status does not appear to distinguish between CD and UC, it is consistently associated with nutritional status (e.g., BMI, albumin, folic acid levels), disease activity (e.g., erythrocyte and leukocyte count, CRP concentration) and severity (e.g., CDAI, HBI, SES-CD). Moreover, emerging data suggest that Se deficiency may contribute to gut dysbiosis, although it is not yet clear whether Se depletion predisposes to IBD or is a consequence of disease activity. Therefore, well-designed clinical trials and multicenter prospective studies involving large cohorts are warranted to clarify these relationships, define the causal role of Se deficiency in IBD development, monitor longitudinal changes in gut microbiota composition and metabolism, and evaluate the potential of Se supplementation as an adjuvant to IBD traditional therapies.

### 4.3. Selenium and Inflammatory Bowel Disease: Oxidative Stress and Inflammation

As previously discussed, Se exerts its biological effects through selenoproteins, which are believed to regulate the progression of IBD via multiple mechanisms. These include antioxidant and anti-inflammatory actions, modulation of intestinal microbiota composition, and contribution to CRC prevention [18,125].

Oxidative stress, arising from an imbalance between the production and clearance of ROS and reactive nitrogen species (RNS), leads to biomolecule oxidation, damage to structural properties of cell membranes, and activation of pro-inflammatory signaling pathways [18,158]. These processes contribute to intestinal injury and are recognized as key drivers in the pathogenesis and development of IBD [18,158]. Prolonged intestinal inflammation is associated with abnormal generation of ROS/RNS, which, by stimulating the production of TNF-α, IL-1β, IL-4, IL-6 and IL-8 within the colon, promote neutrophil infiltration and activation of mitogen-activated protein kinase and nuclear factor kappa-light-chain-enhancer of activated B cells (NF-κB) signaling cascades, processes that compromise epithelial barrier function and exacerbate inflammation, thereby perpetuating a self-regenerating loop (reviewed in [158,159]). In contrast, nuclear factor erythroid 2-related factor 2 (Nrf2) signaling plays a protective role in preserving the mucosal redox balance and function by inducing the transcription of genes involved in antioxidant defense and anti-inflammatory response, cell growth, and survival [158,160]. However, in the context of IBD, persistent oxidative stress promotes Nrf2 upregulation, resulting in Nrf2-dependent stress adaptation of the colonic epithelium and potentially contributing to late damaging and protumorigenic effects [160]. Patients with IBD have a two-to threefold increased risk of developing CRC, which account for 10–15% of IBD-related mortality, while a family history of CRC among first-degree relatives doubles this risk [161,162]. IBD patients are also at elevated risk for extraintestinal malignancies, including cancers of the bile ducts, breast, liver, lung, oral cavity, pancreas prostate, and thyroid, following long-term immunosuppressive therapies [162]. Although CRC incidence in IBD has been steadily declining since 1990, the absolute number of cases is expected to increase in the coming decades due to the increasing global prevalence of IBD [162]. Dual oxidase 2 (DUOX2), an inducible, epithelial-specific NADPH oxidase that interacts with the maturation factor DUOXA2 to produce hydrogen peroxide (H_2_O_2_) from oxygen, representing the predominant system for H_2_O_2_ in the colon, is also upregulated in IBD [163,164]. DUOX2 upregulation is associated with increased abundance of Proteobacteria, contributing to microbiota alterations, chronic inflammation, and heightened CRC susceptibility [164]. Moreover, loss-of-function protein variants in DUOX2 are linked to elevated plasma levels of IL-17C, which has also been detected in mucosal biopsies of IBD patients and is implicated in the activation of specific Proteobacteria pathobionts [163].

Multiple antioxidant defense mechanisms protect cells from excessive ROS/RNS. These include intracellular enzymatic antioxidants (e.g., GPXs, superoxide dismutase—SOD, catalase), intracellular antioxidant molecules (reduced glutathione—GSH), extracellular antioxidants (e.g., tocopherol, ascorbic acid) [158,159]. GPXs, a family of selenoproteins comprising eight isoforms, most of which contain SeCys in their active site, catalyze the reduction of H_2_O_2_ to molecular oxygen and water using GSH as an electron donor, and four expressed in the gut [165,166]. GPX1 is a cytosolic enzyme ubiquitously distributed in all tissues [167]. In a Portuguese cohort comprising 436 CD patients, 367 UC patients, and 434 healthy controls, the AA genotype of rs1050450 variant in *GPX1*, characterized by a 10% lower enzymatic activity, was significantly associated with UC [167]. In contrast, a study conducted on a Polish population (200 IBD patients and 245 controls), found that the C/T and T/T genotypes at codon 197 were non-significantly associated with reduced risk of IBD, suggesting a potentially protective role of this polymorphism [168]. Additionally, in IBD patients in remission, erythrocyte GPX1 activity was significantly higher compared to controls [168]. Therefore, specific amino acid substitutions in GPX1, altering the structural conformation of the protein’s active site, may lead to redox imbalance and contribute to IBD pathogenesis [168].

Unlike GPX1, GPX2 is selectively expressed in the gastrointestinal tract, where its upregulation has been observed both in experimental colitis models and in patients with IBD, suggesting that H_2_O_2_ plays a pivotal role in the inflammatory process underlying IBD and that GPX2 induction serves as a compensatory response to oxidative stress [165,169,170,171]. While mice deficient in either *Gpx1* or *Gpx2* appear phenotypically normal, double-knockout models exhibit symptoms and mucosal inflammation in the ileum and colon, accompanied by elevated levels of lipid hydroperoxides detectable in the colonic mucosa, features consistent with IBD pathology [172]. In dextran sulfate sodium (DSS)-induced colitis, GPX2 expression is enhanced in both the acute and recovery phases of inflammation, driven by IL22–mediated activation of STAT3, a member of the STAT family of cytoplasmic transcription factors involved in cell proliferation, survival and oncogenesis [169,173]. Notably, this regulatory pathway appears to operate independently of Nrf2 signaling [169]. The role of GPX2 in tumorigenesis remains controversial. On one hand, GPX2 suppresses the activity of cyclooxygenase 2 (COX-2), an inducible enzyme responsible for converting arachidonic acid to prostaglandins [174], thereby silencing inflammation [175] and inhibiting tumor cell migration and invasion [176]. On the other hand, Se-deficient GPX2-knockout mice exhibit smaller tumor size compared to the wild-type genotype in chemically induced models of tumors [177,178]. Moreover, GPX2 overexpression has been linked to the formation of differentiated tumor mass and early recurrence of CRC, potentially by counteracting ROS overproduction [179]. Thus, while acknowledging the critical role of GPX2 in regulating inflammation-driven colon carcinogenesis, current evidence suggests the protective role of Se against CRC may be independent of GPX2 expression [177].

GPX3, the only extracellular isoform within the GPX family, functions as a tumor suppressor in the context of inflammatory colon carcinogenesis, likely through its ability to reduce ROS and mitigate DNA damage [180]. In a murine model of azoxymethane (AOM)/DSS-induced colitis, *Gpx3*-deficient mice developed a greater number of tumors and with advanced staging and local invasion, along with enhanced inflammation and increased DNA damage [180]. However, the tumors in *Gpx3*-deficient mice were smaller than those in wild-type counterparts [180]. Indeed, based on its dual role, while GPX3 protects normal cells from oxidative stress and malignant transformation, it promotes tumorigenesis in already transformed cells by shielding them from programmed cell death [180].

GPX4 is the only selenoprotein expressed in three isoforms (cytosolic, mitochondrial and nuclear) and serves as the main oxidoreductase responsible for the clearance of oxidized biolipids, thereby preventing lipid peroxidation [181,182]. Additionally, GPX4 plays a crucial role in inhibiting ferroptosis, a form of Fe-dependent, non-apoptotic cell death, a process which occurs in both cancerous and normal cells [181]. In CD, but not UC, intestinal epithelial cells (IECs) derived from lesional small intestinal mucosa exhibit reduced GPX4 expression and enzymatic activity, accompanied by increased levels of lipid peroxidation, suggesting a disease-specific impairment in lipid redox homeostasis that may contribute to mucosal damage in CD [181]. In a large Czech cohort including 832 CRC cases and 705 controls, three single nucleotides polymorphisms, namely rs713041 (*Gpx4*), rs7579 (*Sepp1*), rs34713741 (selenoprotein S) were significantly associated with a 33–68% increased risk of CRC, and the associations remained significant after adjustment for lifestyle confounders and sex [183]. Notably, risk for CRC was also influenced by two-loci interactions and modulated by sex, although these findings may be confounded by the low Se status of the study population [183].

SEPP1 plays a dual role in Se biology: it transports over 50% of plasma Se owing to its ten SeCys residues per molecule and facilitates Se delivery from the liver to other organs [184,185,186]. In addition to its transport function, SEPP1 acts as a peroxidase, reducing H_2_O_2_ and phosphatidylcholine hydroperoxide with TRX and GSH as electron donors [184,185,186]. Serum SEPP1 concentration is significantly reduced in subjects with CD, but not in those with UC [184]. The decline in SEPP1 biosynthesis may be driven by mucosal inflammation, characterized by elevated levels in IL-1β, TNF-α, and interferon gamma [187]. Besides, CD patients adhering to a long-term elemental diet, which provides minimal Se daily intake (1.6 μg/1000 kcal), exhibit Se levels significantly lower than those on non-elemental diet [184]. Therefore, measuring circulating levels of SEPP1 offers a more effective assessment of Se status and systemic antioxidant activity [184]. The antioxidant function of SEPP1 also suggests its potential involvement in cancer prevention [187]. Mice with *Sepp1* haploinsufficiency or mutations display a higher risk of colitis-associated cancer, linked to impaired oxidative stress response and increased DNA damage [188]. Under these conditions, SEPP1 depletion induces key signaling pathways, including the Keap1/Nrf2 system, central to redox regulation [189], and the Wnt cascade, which governs cell proliferation, differentiation, migration, and tissue homeostasis and whose dysregulation promotes a pro-tumorigenic microenvironment [166,188,190]. Se supplementation in SEPP1-mutated mice significantly improve survival compared to a normal Se diet, underscoring SEPP1’s role in modulating inflammatory-driven tumorigenesis [188]. Notably, unlike partial SEPP1, complete loss of SEPP1 appears to suppress tumor proliferation likely due to enhanced oxidative stress that triggers ROS-dependent ferroptosis, a phenomenon frequently observed in cancer [188].

Furthermore, in a rat model of 2,4,6-trinitrobenzene sulfonic acid-induced colitis, characterized by mitochondrial damage, suppressed respiratory activity, loss of mitochondrial DNA, altered tissue oxygenation, and the development of inflammatory/necrotic conditions, Se supplementation demonstrated protective effects toward colonic mitochondria [191]. Specifically, Se promoted the upregulation of mitochondrial transcription factors required for mitochondrial DNA replication and attenuated neutrophil infiltration, thereby exerting anti-inflammatory and anti-necrotic actions [191].

**Figure 2:**
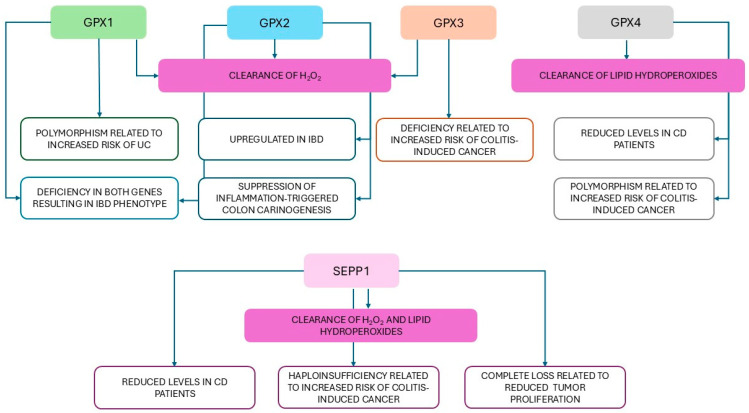
Summary of antioxidant and anti-inflammatory effects of selenoproteins in inflammatory bowel disease. Abbreviations: CD: Crohn’s disease; GPX: glutathione peroxidase 3; H_2_O_2_: hydrogen peroxide; IBD: inflammatory bowel disease; SEPP1: selenoprotein P; UC: ulcerative colitis.

In summary, oxidative and nitrosative stress, coupled with compromised antioxidant systems, are recognized as potent mediators of tissue injuries promoted by inflammation and central contributors to the pathophysiology of IBD (Figure 2). However, the precise mechanisms underlying redox imbalance in IBD need to be deeply elucidated to provide further insights into the disease pathogenesis and shed new light on the therapeutic potential of antioxidant strategies, including Se supplementation, as adjuvant treatments in IBD patients (see Section 4.6).

### 4.4. Selenium and Inflammatory Bowel Disease: Immunity

Se also contributes to the mitigation of IBD by modulating immune responses, particularly through its effects on macrophage activities [18]. Loss of selenoprotein expression in macrophages alters their interaction with extracellular matrix, impairing migration and suggesting a role for Se in regulating macrophage invasiveness and maintaining tissue homeostasis [192]. Se-deficient macrophages also display diminished phagocytic activity and compromised antioxidant defenses, accompanied by an increase in oxidative products and pro-inflammatory cytokines, except for TNF-α, which remains unaffected [193]. As outlined in Section 2, M1 macrophages initiate the inflammatory response by releasing high levels of inflammatory cytokines such as IL-1β, IL-6, IL-12, IL-23 and TNF-α, and nitric oxide, a soluble endogenous gas with various biological functions, including cytotoxic and immunomodulatory properties [18,194,195,196]. In contrast, M2 macrophages, stimulated by Th2 cytokines IL-4 and IL-13, are specialized in promoting the resolution phase of inflammation through the secretion of the anti-inflammatory cytokine IL-10 [195]. Se supplementation in macrophage cultures facilitates the switching from M1 to M2 phenotype in the presence of IL-4 and 15-Deoxy-△^12,14^-prostaglandin (PG) J_2_ (15d-PGJ_2_) [195]. In particular, bioavailability of Se is essential for shunting arachidonic acid metabolism in macrophages from PGE_2_, associated with mucosal injury in UC [197], toward PGD_2_ and its double dehydration product, 15d-PGJ_2_, by regulating the corresponding synthases [198]. Following Se supplementation, 15d-PGJ_2_ promotes resolution of intestinal colitis by reducing the number of neutrophils and M1 macrophages [199,200] and by activating the peroxisome proliferator-activated receptor gamma (PPARγ), a nuclear hormone receptor involved in energy metabolism and pathways related to lipid and glucose homeostasis and anti-inflammatory signaling [27,195,201]. PPARγ, widely expressed in colonic epithelial cells and, to a lesser extent, in macrophages and lymphocytes, functions as a transcriptional regulator and through DNA-independent patterns with other transcriptional factors [202]. Upon activation, PPARγ inhibits NF-κB signaling pathway and dampens gut inflammation by modulating the release of inflammatory mediators and favoring Tregs recruitment during the later stage of experimental IBD [203]. Conversely, PPARγ deletion in macrophages exacerbates disease activity [204]. Consistently, PPARγ is downregulated in active UC and its expression inversely correlates with disease activity, underscoring its critical role in UC pathogenesis [202,205,206].

GPX2 can also directly inhibit the NF-κB pathway, which plays a key role in innate immune activation and whose dysregulation contributes to the pathogenesis of various inflammatory diseases, including IBD [207,208]. Experimental evidence from both in vivo and in vitro studies demonstrates that loss of GPX2 leads to upregulated expression of inducible NO synthase (responsible for the synthesis of NO under inflammatory condition [209]), as well as increased levels of TNF-α and COX-2, all of which are transcriptionally regulated by NF-κB [207]. Interestingly, the upregulation of these pro-inflammatory mediators is higher in GPX2 than in GPX1 knockdown models, whereas the elevation of pro-inflammatory COX2-derived prostaglandin PGE_2_ (a lipid mediator known to promote Th17 differentiation via IL-23 induction [27]), is more pronounced in GPX1-deficient cells [207]. These findings indicate that, despite the overlapping functions of GPX1 and GPX2 in attenuating inflammation, they may differently regulate specific components of the inflammatory cascade [207].

Macrophages display considerable heterogeneity and plasticity, adapting their phenotype in response to microenvironmental stimuli [210]. While during the initial stages of tumor development, M1 macrophages exert antitumoral effects, as tumors progress, these cells tend to polarize toward the M2 subtype, which promotes tumor growth, supports angiogenesis, and facilitates tumor cell migration, features that make M2 prevalence a prognostic marker in cancer [210]. In the AOM/DSS murine model of inflammatory carcinogenesis, *Gpx3* loss is associated with a reduction in M1 macrophages and a concomitant increase in M2 macrophages [180,210]. Similarly, tumors from AOM/DSS-treated mice with haploinsufficiency for *Sepp1* exhibit an increase in M2 polarized macrophages alongside diminished expression of the M1 markers, collectively contributing to tumorigenesis [188]. Therefore, both GPX3 and SEPP1 may influence intratumoral macrophage composition and function, thereby serving as tumor suppressors in colitis-associated cancer models [188].

Selenoproteins are also involved in T cell development and activation [211]. Mice lacking selenoproteins in T cells exhibit impaired maturation of competent T cells and defective T cell-mediated immune responses [211]. Selenoprotein deficiency causes overproduction of ROS upon T cell receptor stimulation, which inhibits T cell proliferation and activation [192,211]. Ablation of *Gpx1* in Th cells results in elevated levels of ROS and IL-2 compared to wild-type Th cells, promoting their proliferation at a faster rate than normal Th cells and differentiation into Th1 rather than into Th2 or Th17 cells, which underscores the pivotal role of ROS in driving Th cell fate [212]. Deletion of *Txnrd1*, the gene encoding TRX reductase-1, a key component of the cytosolic antioxidant TRX1 system together with TRX and its negative regulator thioredoxin-interacting protein (TXNIP), impairs T-cell proliferation during thymic development [213]. This is due to TRX-1’s essential function at the last step of t nucleotide biosynthesis, where it transfers electrons to ribonucleotide reductase [213]. Conversely, genetic deletion of *Txnip* does not lead to uncontrolled proliferation of T and B lymphocytes, although TXNIP suppresses T and B cell expansion and prevents their hyperproliferation [214].

Selenoprotein W (SELW), whose function remains only partly elucidated, is also implicated in T cell regulation [215]. SELW deficiency is associated with impaired CD4+ T cell proliferation and blockage of the inhibitory effect of selenite on Th1 differentiation [215]. Se supplementation suppresses Th1 polarization, a hallmark of CD, via both SELW-mediated ROS scavenging and inhibition of one carbon metabolism, a folate-dependent pathway essential for the novo synthesis of purines in T cells [215,216].

**Figure 3:**
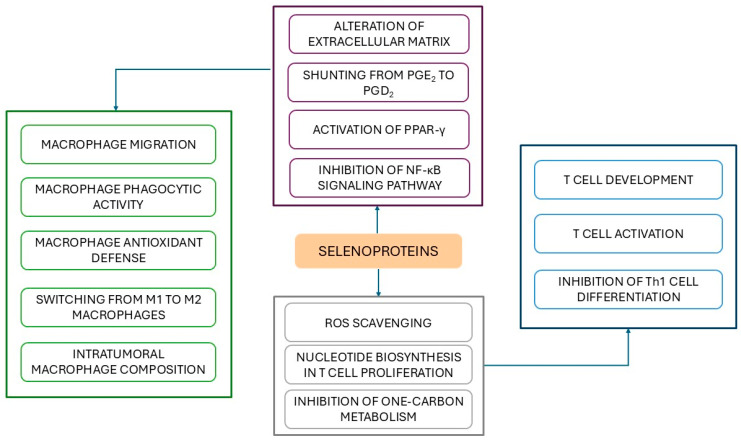
Actions of selenoproteins on the immune system in the context of inflammatory bowel disease. Abbreviations: NF-κB: nuclear factor kappa-light-chain-enhancer of activated B cells; PG: prostaglandin; PPAR-γ: peroxisome proliferator-activated receptor gamma; ROS: reactive oxygen species; Th: T helper.

Collectively, these data highlight the multifaceted and beneficial effects of Se and selenoproteins in modulating both innate and adaptive immune responses in IBD and involving specific metabolic processes, which offer promising targets for therapeutic interventions (Figure 3). Future research should consider the influence of other critical determinants such as dietary patterns and environmental factors, which may further shape this complex immunometabolic system.

### 4.5. Selenium and Inflammatory Bowel Disease: Interactions with Gut Microbiota

Although Se is one of the most common trace elements in the human diet, relatively few studies have investigated its effects on the gut microflora composition [217]. Similar to eukaryotes, approximately 25% of prokaryote genomes encode selenoenzymes, including formate dehydrogenases in *Escherichia coli* and *Campylobacter jejuni*, glycine reductases in a variety of anaerobic microorganisms including *Eubacterium acidaminophilum* and various *Clostridium* species, and xanthine dehydrogenase in the opportunistic pathogen *Enterococcus faecalis* [217]. Since the intestinal microbes can use dietary Se to synthesize their own selenoproteins, changes in dietary Se may influence the composition and diversity of the gur microbial community [132]. Experimental data indicate that dietary Se enhances microbiota diversity, with the most pronounced effects in Bacteroidetes, although responses depend on phylotype [218]. More modest changes are seen in Firmicutes, regardless mice are conventionally raised or germ-free [218]. Another study found that, while Se intake modulates microbial composition, it has no significant impact on the richness of microorganisms [219]. In particular, Se-deficient diet was associated with a non-significant increase in *Dorea* spp., a hydrogen and carbon dioxide producer found at elevated levels in patients diagnosed with irritable bowel syndrome and multiple sclerosis [219,220,221]. Supranutritional Se supplementation in mice led to a significant increase in *Turicibacter*, a genus with anti-inflammatory properties whose depletion is linked to colitis and reduced levels of butyrate, and *Akkermansia*, a SCFA producer that enhances mucus secretion and preserves intestinal barrier integrity [219,222,223]. Additionally, mice colonized with fecal microbiota from deficient Se-supplied donors exhibited more severe symptoms of DSS-induced colitis compared to mice transplanted with microbiota from donors with supranutritional Se supplementation [219]. This result underlines the protective effects of Se-adequate microbiota transplantation in maintaining intestinal health and mitigating experimental colitis [219].

On the other hand, bacteria may compete with the host for Se under conditions of limited micronutrient availability [224]. Germ-free mice do require less Se for selenoprotein synthesis than conventionally colonized animals, as reflected by increased tissue expression and activity of GPX and TRX reductase, along with higher Se concentration in plasma, liver, and intestinal tissue, indicating that microbial colonization may increase the host susceptibility to Se deficiency [224]. In case of inadequate Se intake, Hridina et al. [224] estimated that host selenoprotein levels may drop by two- to threefold, potentially leading to adverse health outcomes. Likewise, germ-free mice exhibit elevated levels of stress-related selenoproteins GPX1 and methionine-R-sulfoxide reductase 1 in the liver and kidneys, indicating that gut microorganisms may limit Se availability for the host independently of other micronutrient status [218].

To date, only one study has directly examined the interaction between Se intake, gut microbiota composition, and IBD. In a Chinese case–control study [225] involving 280 patients with IBD (UC: *n* = 107; CD: *n* = 173) and 42 healthy controls, the mean daily intake of Se in the IBD group (38.8 ± 28.8 µg in UC patients; 39.25 ± 22.17 µg in CD patients) was significantly lower compared to controls (51.09 ± 31.94 µg). The α-diversity of the gut microbiota was significantly lower in CD patients than in healthy individuals [225]. When comparing metrics in fecal and mucosal samples, α-diversity was significantly lower in fecal samples across both UC and CD groups [225]. Furthermore, bacterial richness differed profoundly between the two sample types in both groups, with mucosal samples containing approximately ten times more abundant genera than fecal samples [225]. In particular, the mucosal microbiota of UC and CD patients was enriched, respectively, with 50 and 63 genera, respectively, belonging to the phylum Proteobacteria, whose expansion has been implicated in IBD pathogenesis and experimental colitis [225,226,227]. Diet-microbiota interaction analysis revealed 22 and 37 microbial species correlated with diet and metabolites in UC and CD groups, respectively [225]. In CD patients, Se intake showed a negative correlation with *Shigella*, anaerobic bacteria associated with diarrhea and dysentery [228], and a positive correlation with *Sporobacter*, a genus linked to improvement of chronic colitis [225,229].

In sum, these findings indicate a bidirectional relationship between Se and the gut microbiota. Dietary Se intake influences microbial composition and diversity, substantially impacting on intestinal barrier integrity and susceptibility to inflammatory diseases such as IBD. Conversely, Se supplementation may represent an effective method for improving dysbiosis in IBD patients and enriching beneficial microbiota taxa, which are essential for intestinal health.

### 4.6. Selenium and Inflammatory Bowel Disease: New Frontiers of Treatment

As observed, in cases of nutritional deficiency, Se supplementation can be beneficial for individuals affected by UC particularly when IECs are damaged [230]. In this context, elemental Se appears to be less toxic than Se-based compounds such as selenate, selenite, and selenide, and shows improved biocompatibility when delivered as Se nanoparticles (SeNPs) [230]. The use of nanoparticles (NPs), typically ranging from 1 to 100 nanometers, for the delivery of bioactive compounds represents an innovative and transformative technology, enabling enhanced drug absorption and improved nutrient bioavailability [231,232]. Indeed, NP surfaces can be functionally modified with targeting ligands, allowing for more precise drug delivery and, consequently, greater therapeutic efficacy [232].

SeNPs, produced by inorganic Se through physical, chemical, or biological methods, have demonstrated remarkable versatility owing to their size, surface charge, and controlled-release properties [230]. Moreover, they exhibit increased stability, lower toxicity and reduced excretion compared to inorganic Se forms and organic selenocompounds [231,233]. Over the past decade, SeNPs have been extensively investigated for their use as dietary supplements and antioxidants, while also exhibiting cytotoxic properties with potential applications in cancer therapy [234].

Although chemical synthesis—typically employing sodium selenite, selenious acid, or selenium dioxide as precursors in combination with reducing agents—is the most widely used method for preparing SeNPs, the resulting NPs are inherently unstable and prone to aggregation [235]. Surface functionalization mitigates non-specific interactions with biological components, thereby enhancing both the stability and bioavailability of SeNPs [235]. In particular, SeNPs can be functionalized with *Eucommia* ulmoides polysaccharide (EUP), a natural polysaccharide, to form EUP-SeNP [236]. EUP-SeNPs ameliorate the symptoms of DSS-induced colitis in murine models [236]. Treated mice exhibit improved intestinal permeability and enhanced barrier integrity, alongside increased antioxidant capacity, reduced levels of apoptosis and inflammation [236]. Interestingly, EUP-SeNPs also appear to regulate intestinal microbiota composition by promoting the growth of beneficial taxa (e.g., Actinobacteriota, Deferribacterota, Rikenellaceae, Muribaculaceae) and decreasing the abundance of pathogenic microflora (e.g., Campylobacterota, Clostridia, Oscillospirales, Desulfovibria, Ruminococcaceae) [236]. Additionally, EUP-SeNP treatment can effectively inhibit lipopolysaccharide-induced activation of the Toll-like receptor-4/NF-κB signaling pathway in IEC lines, suggesting their potential use in the clinical treatment of IBD [236].

Low molecular weight chitosan selenium nanoparticles (LCS-SeNPs), a novel formulation of SeNPs with an average diameter of 198 nm, significantly promote weight gain in mice undergoing DSS treatment and reduce inflammatory cell infiltration [237]. Furthermore, LCS-SeNPs markedly decrease serum IL-6 and TNF-α, counteract the DSS-induced upregulation of Nrf2, and enhance serum GPXs levels, collectively contributing to a substantial reduction in intestinal damage [237].

In such a framework, Yang and collaborators [230] put a step forward into the state-of-the art preparing a system based on mannose-SeNPs modified through the ascorbic acid reduction of Na_2_SeO_3_ with mannose supplementation. Notably, the combination of the well-documented anti-inflammatory properties of the hexose sugar mannose with functionalized SeNPs leads to increased expression of GPXs and modulates the physiological response of IECs [230]. Additionally, mannose-SeNPs have been shown to attenuate oxidative stress and inflammation by inhibiting the NF-κB signaling in the colon, thereby alleviating colitis-related symptoms in a DSS-induced colitis model [230].

SeNPs exhibit multifunctional properties in targeted delivery and controlled release, and, due to their high bioavailability, low toxicity, and cost-effectiveness, they represent one of the most promising forms for supplementation [238]. However, successful clinical translation of SeNPs requires comprehensive preclinical studies in animal models to evaluate pharmacokinetics, long-term safety, and potential off-target effects [238].

## 5. Conclusions and Future Perspectives

Se has been universally recognized as an essential element due to its diverse physiological functions, although its efficacy is confined to a narrow range of plasma concentration. In the context of IBD, a multifactorial condition influenced by host genetics, immune dysregulation, gut microbiota and environmental determinants, and whose global prevalence is steadily increasing, the role of diet and micronutrients, such as vitamins and trace elements, has gained increasing attention. Excessive production of ROS/RNS, coupled with impaired antioxidant defense, is a critical factor in IBD progression and severity. Despite limitations, most epidemiological studies report reduced Se levels in IBD patients, with an inverse relationship between Se status and disease severity. This highlights the need for deeper investigation into the molecular mechanisms linking Se to IBD pathogenesis. Selenoproteins, in particular, show promise in mitigating oxidative stress, modulating inflammation, and interacting with immune cells and gut microbiota, making them potential targets for novel therapeutic strategies. Nonconventional approaches to IBD treatment such as inhibitors of ROS generation (e.g., inhibitors of cyclooxygenase, angiotensin II type 1 blockers, statins), hormones (e.g., melatonin), synthetic substances (e.g., N-acetylcysteine, recombinant SOD), and natural compounds such as polyphenols (e.g., berberine, curcumin, resveratrol, quercetin), micronutrients (e.g., omega-3 fatty acids, vitamins A, C, E, carotenoids, Se, Cu, Zn), prebiotics, probiotics, and postbiotics have demonstrated efficacy in reinforcing the immune function, reducing excess of reactive species, and alleviating mucosal inflammation in both experimental models and clinical trials [167,174]. Given the broad-spectrum of Se-related functions and the large number of identified selenoproteins—some of which remain functionally uncharacterized—further research is essential to elucidate additional mechanisms through which Se exerts beneficial effects in IBD. Although only a limited number of clinical studies have investigated the therapeutic effects of Se in IBD, one trial demonstrated that oral selenomethionine capsules administered at a dosage of 200 mcg/day for ten weeks in patients with active mild-to-moderate UC led to the restoration of adequate Se levels, reduced inflammation, and overall clinical improvement [239]. However, multicenter clinical trials with longer intervention periods and varying Se dosages are needed to confirm these findings and to define the optimal Se regimen capable of promoting clinical remission without adverse effects. In this context, both genetic factors and the microbiome drive the individual’s differentiated response to Se supplementation in the humans and in the living beings in general [240]. Therefore, integrating pharmacogenomic and pharmacomicrobiomic approaches could optimize treatment outcomes and enhance overall safety, effectively putting into practice one of the key foundations of personalized medicine. Furthermore, the promising rise in new materials and production means enabled researchers to synthetize Se-based nanoparticles, particularly useful in the framework of specific compounds’ delivery, whose employment is gaining momentum and whose advantages are tangibles, at least in animal models. Although their translation in clinical practice is still in its infancy and requires careful investigations, their undoubtedly positive results in preliminary investigations would make their large-scale adoption just a matter of time to the benefits of patients suffering from IBD and similar conditions.

## Figures and Tables

**Table 1:** Key features of the major susceptibility genes for inflammatory bowel disease.

Acronym	Full Name	Actions on the Immune System	Actions on the Microbiota	References
*NOD2*	Nucleotide-binding oligomerization domain-containing protein 2	Involvement in the innate immune response; induction of autophagy in dendritic cells	Gene deficiency or mutations associated with increase in *Escherichia coli* and decrease in *Faecalibacterium prausnitzii*	[68,70,71]
				
*ATG16L1*	Autophagy-Related 16 like 1	Induction of autophagy in Paneth cells	Gene mutations related to the increase in Bacteroidetes, Proteobacteria, and Cyanobacteria and decrease in Firmicutes	[72,73,74]
				
*CARD9*	Caspase Recruitment Domain 9	Most variants (rs10870077, rs4077515, rs10781499) enhancing immune response	Modulation of gut microbiota balance by increasing the levels of Firmicutes and reducing the levels of Clostridiaceae. *CARD9* rs10781499 associated with dysregulation of tryptophan metabolism	[30,75,76]
*TNFSF15*	Tumor Necrosis Factor Superfamily Member 15	Intracellular bacterial clearance; when highly expressed, induction of IL-2, IL-4; IL-13, IFN-γ secretion	-	[77,78]

Abbreviations: IFN-γ: interferon gamma; IL: interleukin.

**Table 2:** Clues and pitfalls in the relationship between selenium status and inflammatory bowel disease.

Clues	References	Pitfalls	References
Significantly lower blood concentration in CD/UC patients compared to healthy controls	[19,28,153,157]	Single-center study	[19,153,154,156,157]
			
Significant correlations of serum Se concentration with CD/UC activity-related parameters	[19,153,154,156,157]	Small sample size	[153,154,156,157]
			
Serum Se concentration significantly inversely correlated with CD/UC severity	[154,156,157]	Results potentially affected by geographic region, sample size, and/or dietary factors	[154]
			
Low blood Se concentration in CD patients during clinical remission	[155]	Inconsistent evidence on significant differences in blood Se levels between CD patients in clinical remission and healthy controls	[155]
			
Se deficiency significantly correlated with disease exacerbation in UC patients	[19]	No significant association between blood Se levels and CD activity	[155]
			
Se deficiency significantly correlated with alterations in diversity and composition of the gut microbiota in CD patients	[155]	Variability between studies in the cut-off, units and statistical methods	[155]
		No adequate exploration of the cause of Se deficiency	[155]
			
		Possibility of publication bias	[28]
			
		Absence of standardized diagnostic criterion for IBD across different countries and study periods	[28]

Abbreviations. CD: Crohn’s disease; IBD: inflammatory bowel disease; Se: selenium; UC: ulcerative colitis.

## Data Availability

No new data were created.

## Abbreviations

The following abbreviations are used in this manuscript:

BMI	Body mass index
CD	Crohn’s disease
CDAI	Crohn’s disease activity index
COX	Cyclooxygenase
CRC	Colorectal cancer
CRP	C reactive protein
DC	Dendritic cell
DSS	Dextran sulfate sodium
DUOX2	Dual oxidase 2
EUP	*Eucommia* ulmoides polysaccharide
FCP	Fecal calprotectin
GPX	Glutathione peroxidase
GSH	Reduced glutathione
H_2_O_2_	Hydrogen peroxide
H_2_S	Hydrogen selenide
HBI	Harvey-Bradshaw index
IBD	Inflammatory bowel disease
IEC	Intestinal epithelial cell
LCS-SeNP	Low molecular weight chitosan selenium nanoparticles
NOD	Nucleotide-binding oligomerization domain
NP	Nanoparticle
Nrf2	Nuclear factor erythroid 2-related factor 2
NF-κB	Nuclear factor kappa-light-chain-enhancer of activated B cells
PG	Prostaglandin
PPARγ	Peroxisome proliferator-activated receptor gamma
RNS	Reactive nitrogen species
ROS	Reactive oxygen species
SCFA	Short chain fatty acid
Se	Selenium
SeCys	Selenocysteine
SeNP	Selenium nanoparticle
SES-CD	Simple endoscopic score for Crohn’s disease
SOD	Superoxide dismutase
Th	T helper cell
TNF-α	Tumor necrosis factor alpha
Treg	Regulatory T cell
TRX	Thioredoxin
UC	Ulcerative colitis
